# Time and Narrative: An Investigation of Storytelling Abilities in Children With Autism Spectrum Disorder

**DOI:** 10.3389/fpsyg.2018.00944

**Published:** 2018-06-19

**Authors:** Francesco Ferretti, Ines Adornetti, Alessandra Chiera, Serena Nicchiarelli, Giovanni Valeri, Rita Magni, Stefano Vicari, Andrea Marini

**Affiliations:** ^1^Cosmic Lab, Department of Philosophy, Communication and Performing Arts, Roma Tre University, Rome, Italy; ^2^Child and Adolescent Neuropsychiatry Unit, Department of Neuroscience, The Bambino Gesù Children’s Hospital, IRCCS, Rome, Italy; ^3^Department of Languages and Literatures, Communication, Education and Society, University of Udine, Udine, Italy; ^4^Claudiana - Landesfachhochschule für Gesundheitsberufe, Bozen, Italy

**Keywords:** autism spectrum disorder, episodic future thinking, global coherence, mental time travel, narrative

## Abstract

This study analyzed the relation between mental time travel (MTT) and the ability to produce a storytelling focusing on global coherence, which is one of the most notable characteristics of narrative discourse. As global coherence is strictly tied to the temporal sequence of the events narrated in a story, we hypothesized that the construction of coherent narratives would rely on the ability to mentally navigate in time. To test such a hypothesis, we investigated the relation between one component of MTT—namely, episodic future thinking (EFT)—and narrative production skills by comparing the narratives uttered by 66 children with high-functioning autism spectrum disorder (ASD) with those produced by 66 children with typical development. EFT was assessed by administering a task with minimal narrative demands, whereas storytelling production skills were assessed by administering two narrative production tasks that required children to generate future or past episodes with respect to the target stimuli. The results showed that EFT skills were impaired only in a subgroup of children with ASD and that such subgroup performed significantly worse on the narrative production task than ASD participants with high EFT skills and participants with typical development. The practical and theoretical implications of these findings are discussed.

## Introduction

The ability to tell stories is a species-specific feature of human beings. Niles (1999) defined individuals of our species as *Homo narrans*. In a similar way, Corballis (2015, p. 107) maintained that “if there is anything that defines our species as unique… it is the telling of stories, and the invention of language as the means of doing so”. Indeed, according to several scholars, the ability to tell stories represents the evolutionary adaptation that distinguishes humans from other animals (Sugiyama, 2001; Thompson, 2010; McBride, 2014; Adornetti, 2015; Ferretti, 2016; Corballis, 2017; Ferretti et al., 2017).

Defining what characterizes a narrative is a matter of controversy. There is a general agreement on the fact that narratives do imply a reference to sequences of events in time (Scalise-Sugiyama, 2005). In the present study, we focused on a specific property of narrative and on a specific cognitive ability. *Global coherence* is the narrative property called upon when constructing the plotline necessary to process the gist of a story. The specific cognitive ability—*Mental Time Travel* (MTT)—is the skill that allows humans to navigate in time (Suddendorf and Corballis, 1997, 2007). From a theoretical point of view, since global coherence is a property strictly tied to the temporal sequence of events in a story, we argue that the processing of that story is largely dependent on the individual’s ability to navigate in time. From an empirical point of view, our aim was to investigate the role of MTT in narrative by analyzing storytelling in children with autism spectrum disorder (ASD), a neurodevelopmental disorder characterized by deficits in social communication and interaction, along with restricted repetitive interests and behaviors (American Psychiatric Association [APA], 2013). Children with ASD are reported to have difficulties in narrative, especially in respect to qualitative aspects, such as the gist of the story and the organization of coherent chains of events (see section “Narrative Global Coherence as the Construction of Causal Chains”). Furthermore, children with ASD have been reported to show impairments in managing the temporal dimension of experience (see section “Episodic Memory and Episodic Future Thinking in Autism”).

In the past, several scholars have supported the idea that the triad of behavioral impairments characterizing ASD—social interaction, communication, and imaginative flexible functions—is attributable to a single cause, although there has been disagreement as to what that cause might be. According to the *fractionable triad of autism model* (Happé et al., 2006; Happé and Ronald, 2008) the three diagnostic domains of ASD have independent causes at the genetic, cognitive, and neural levels. The cognitive level is particularly relevant to account for the narrative abilities of individuals with ASD. In this respect, the triad of behavioral impairments characterizing ASD has been interpreted as reflecting a deficiency in three cognitive domains: an impairment in theory of mind (e.g., Baron-Cohen et al., 1985; Perner et al., 1989), a problem in central coherence (e.g., Jolliffe and Baron-Cohen, 1997; Happé et al., 2001), or a dysfunction in executive processes (e.g., Ozonoff, 1995; Robinson et al., 2009). Recently, King et al. (2014) have suggested that the cognitive systems involved in the triad of behavioral impairments are all engaged in the narrative deficits of individuals with ASD (see Baron-Cohen, 1988; Bruner and Feldman, 1993; Jolliffe and Baron-Cohen, 2000; Zalla et al., 2006; Nuske and Bavin, 2011; Barnes and Baron-Cohen, 2012).

In this current study, we endorse the idea that the narrative skills of children with ASD should be explained with reference to multiple cognitive processes. That said, our proposal is an integration of those models centered on the role of three single cognitive domains: in addition to the role of theory of mind, central coherence and executive functions, we suggest the need to investigate the narrative deficits of individuals with ASD also considering the crucial role of the cognitive ability that allows individuals to travel in time. More specifically, our hypothesis is that the proper functioning of MTT represents a constituent condition for the generation of coherent narratives. To this extent, we propose that the narrative deficits of individuals with ASD might be further investigated in reference to a potential impairment of MTT.

## Theoretical Background

### Narrative Global Coherence as the Construction of Causal Chains

Scholars widely share the idea that global coherence is at the base of the construction of relationships among events that represent a story’s structure. Affirming that global coherence guides narrative means that the gist of a story is closely tied to the network of causal connections that link the events characterizing different episodes of a story. In line with this hypothesis, Sah and Torng (2015, p. 2) define *global coherence* as the “global representation of story meaning and connectedness,” and Jolliffe and Baron-Cohen (2000, p. 1169) regard global coherence as “the ability to establish causal connections and interrelate local chunks into higher-order chunks so that most linguistic elements are linked together thematically.”

What kind of causal connections are involved in global coherence? Several studies have analyzed stories produced by people with ASD using the causal network model (CNM) proposed by Trabasso et al. (1984), Trabasso and Sperry (1985), Trabasso and Van Den Broek (1985). At the basis of the CNM are two kinds of causal relationships: causal connections and causal chains. Causal connections have a local character: they refer to the overtly or non-overtly marked causal relations between pairs of narrative events. On the contrary, the causal chains among events have a global character, as they relate to utterances connected by causes and consequences during the development of the story (they consist of a sequence of events that form the gist of a story) (Trabasso and Sperry, 1985). According to the CNM, it is mostly through causal chains that a story’s global meaning is given coherence as the global meaning of a narration implies the processing of wide connections between single pieces of information (Jolliffe and Baron-Cohen, 2000). From this view, global coherence is a qualitative property characterized by a holistic character not reducible to local connections between pairs of events (Cosentino et al., 2013).

Consistent with the framework of investigation proposed by the CNM, several studies have suggested that the impairments in storytelling of individuals with ASD can be explained with reference to a specific difficulty in identifying the causal network of a narrative structure (Losh and Capps, 2003; King et al., 2013, 2014; Sah and Torng, 2015). Applying the CNM to the study of narrative in this population, Diehl et al. (2006) reported that children with ASD obtained lower measures of causal connections than children with typical development. However, contrary to expectations, Diehl et al. (2006) as well as Sah and Torng (2015) noted that children with ASD and children with typical development were equally sensitive to the causal-chains events of a story. From these results, it seems that the global narrative impairments reported in ASD cannot be explained with reference to the CNM. It is our proposal that such a model might be extended to include the role of further aspects in addition to the causal connections between events of a story. Indeed, since narrative processing is governed by the global level involved in connecting not adjacent events, namely events that are distant in time, a crucial role in processing coherent narratives might be ascribed to a factor completely omitted by the CNM: the time factor. In the next section, we will take into account such a factor by introducing a different theoretical model.

### Acknowledgment of the Time Factor in Narrative Processing

Jolliffe and Baron-Cohen (2000) have linked the narrative deficits of global coherence in individuals with ASD to their impairments of central coherence – a tendency to focus on details without being able to integrate them into a wider global context (see also Nuske and Bavin, 2011). In their experiments, the authors tested two conditions: (1) a temporal condition in which subjects were asked to reconstruct the correct sequence of events in a story containing markers of time and (2) a coherence condition in which subjects were asked to reconstruct the correct sequences of events in a story without temporal markers. The authors suggested that the more appropriate way to investigate the narrative abilities of individuals with ASD is by excluding the temporal condition, a condition that the authors consider unimpaired. While the principle is methodologically sound, a procedure of this kind entails two consequences. The first is corroborating the idea that the representation of time is not involved in the mental operations necessary to construct global coherence. The second is taking for granted that the temporal dimension of experience is fully accessible for people with ASD because of their ability to use the temporal markers. However, both these consequences are highly controversial. In this final section we show why the representation of time appears to be necessary for the construction of global coherence. In the Section “Episodic Memory and Episodic Future Thinking in Autism” we will address the issue of time representation in individuals with ASD.

Currently, it is a much-debated question whether the temporal dimension should be considered a condition of global coherence. Giora and Shen (1994) maintained that the processing of narrative requires the ability to analyze “a higher-order organization which hierarchically connects not only adjacent events… but also events which are remote from one another on the *temporal axis* of a given discourse” (p. 450 emphasis ours). Karmiloff-Smith (1985, p. 62) suggested the idea that global coherence is the temporal/causal structure of story content. Consistent with these considerations, we suggest that the time factor represents an important aspect in the construction of global coherence. At the basis of our hypothesis are some general theoretical issues. Herman (2013), a scholar involved both in narratology and cognitive science, referred to the narratological tradition inspired by the work of Genette (1972; for subsequent developments, see Ireland, 2001; Bridgeman, 2005; Matz, 2011) and of Ricoeur [developed by Bruner (1999, 1991) and cultural psychologists] to propose a clear idea for reflection: the intrinsically temporal nature of narrative. Namely, Herman (2013, p. 301) argued that “stories… are a primary technology for making sense of how things unfold in time.” Similarly, Abbott (2008, p. 3) suggested that “narrative is the principal way in which our species organizes its understanding of time.” According to Corballis (2015) the temporal character of narrative pertains to the fact that telling a story implies a detachment from the present and a projection in a time different from the here and now. In his view, the same constructive process that allows human beings to reconstruct the past and construct possible futures also allows them to invent stories (Corballis, 2011). Following this view, our idea in this current study is that among the cognitive abilities underlying narrative coherence, we must recognize the crucial role of temporal navigation that allows people to project themselves backward and forward in time.

### Episodic Memory, Episodic Future Thinking and Their Relationships

As mentioned in the Introduction, mental time travel (MTT) is the cognitive system that allows individuals to project backward and forward in time (Tulving, 1985, 2001, 2005; Suddendorf and Corballis, 1997, 2007). It is composed of two closely related abilities: the ability to remember past experiences or *episodic memory* (EM; Tulving, 1985, 2005) and the ability to imagine possible future experiences, called *episodic future thinking* (EFT; Atance and O’Neill, 2001, 2005; Schacter et al., 2017). EM allows individuals to re-experience events from their own past, while EFT allows them to pre-experience events from their own potential future. In this sense, MTT is an ability that does not merely reflect the extraction from memory of a specific meaning or knowledge; rather, it involves the retrieval of previously experienced episodes, as well as the generation of potential future ones (Schacter and Addis, 2007).

A number of studies have recognized the existence of important connections between EM and EFT. Neuropsychological investigations have shown that patients with impairments in EM have difficulties also in envisioning future events (e.g., Klein et al., 2002). Neuroimaging studies on healthy subjects showed that many of the same brain regions—medial temporal and frontal lobes, posterior cingulate, and retrosplenial cortex, as well as lateral, parietal, and temporal areas—are active both when remembering the past and envisioning the future (Addis et al., 2007, 2009; Buckner and Carroll, 2007; Hassabis et al., 2007). Furthermore, developmental research has found that EM and EFT emerge in tandem between 3 and 5 years of age (Atance and Meltzoff, 2005; Busby and Suddendorf, 2005; Hayne et al., 2011; Scarf et al., 2013; Atance and Sommerville, 2014).

Based on the commonalities between remembering the past and envisioning the future, several hypotheses have been proposed regarding the nature of MTT. Schacter and Addis (2007) suggested the *constructive episodic simulation hypothesis*. According to this hypothesis, EM supports future simulation (construction of a specific mental representation of the future) by allowing people to flexibly extract and recombine elements of past experiences to generate novel scenarios. An important aspect of this hypothesis is that remembering the past and imagining the future both involve constructive processes. Indeed, Schacter and Addis (2007) noted that EM does not function in a reproductive manner, similar to a video recorder; rather it is vulnerable to errors and distortions. From this point of view, EM involves conscious acts of construction. In the same way, since the future is not an exact replication of the past, future episodes may require a system of construction rather than reproduction. Buckner and Carroll (2007) focused on the prospection component, suggesting that *self-projection* could be the crucial common process of both EM and EFT: the shifting perception of oneself from the immediate environment to an alternative and imagined future one. Hassabis and Maguire (2007), while acknowledging the importance of self-projection for episodic memory recall and thinking about the future, suggested that functions not explicitly connected to the Self (e.g., imagining fictitious experiences) can better explain commonalities in the brain areas activated by EM and EFT tasks. Accordingly, they proposed that both EM and EFT rest on the capacity for *mental scene construction*, which refers to the generation and maintenance of coherent, multimodal spatial representation. Specifically, scene construction involves binding multiple elements of an imagined scene, such as feelings, thoughts, people, and objects. Thus, according to Hassabis and Maguire (2007, 2009) remembering/foreseeing one’s own past/future and remembering/foreseeing someone else’s past/future both rely on a similar scene construction process. Similarly, Perrin and Michaelian (2017; see also Michaelian, 2016) maintained that EM and EFT do not necessarily involve autonoetic consciousness (i.e., the preservation of the subject’s experience of the remembered event) as both are simulational processes that might concern either oneself or another. This point is key to our proposal since we suggest that MTT is involved not only in the construction of personal narratives, but also in the construction of narratives detached from any personal experience and which instead involve events of another (both fictitious or real) person.

### Episodic Memory and Episodic Future Thinking in Autism

In the past few years, it has been argued that ASD offers an ideal test to unveil the nature of MTT (Lind and Williams, 2012) given that it is a neurodevelopmental disorder characterized by deficits in autobiographical memory, which largely relies on EM (Klein et al., 1999; Bowler et al., 2000; Goddard et al., 2007; Crane and Goddard, 2008). Considering this and based on the commonalities between EM and EFT, several studies have begun exploring the possibility that imagining the future might be impaired in people with ASD (Jackson and Atance, 2008; Lind and Bowler, 2010; Terrett et al., 2013; Hanson and Atance, 2014; Marini et al., 2016). Lind and Bowler (2010) interviewed participants and asked them to detail events from a range of periods (from “today” to “10 years ago/in 10 years”). Results showed that adults with ASD recalled/envisioned fewer specific events than did typical adults, and both groups performed better in recalling past events as opposed to simulating future ones. Terrett et al. (2013), who tested EFT in a group of children with high-functioning ASD, obtained similar results. Marini et al. (2016) assessed self-based and mechanical-based future thinking in children with ASD: in tasks assessing self-based future projection, children were asked to project themselves into the near future. Mechanical-based tasks assessed the children’s ability to predict the outcome of a physical transformation that did not imply any projection of the Self. Results showed that children with ASD had impaired EFT in both kinds of tasks as well as more difficulty performing self-based tasks than mechanical ones. However, it is worth noting that available experimental findings are not entirely consistent. For example, Crane et al. (2013) assessed EM and EFT in ASD subjects and achieved different outcomes. They asked participants to complete a sentence aimed at eliciting past (e.g., “I still remember well how…”) and future event descriptions (e.g., “Next year, I…”). The results showed no group differences in either past or future event conditions.

Overall, the results on EFT skills in people with ASD are far from conclusive. The available investigations often included few participants (see Jackson and Atance, 2008; Crane et al., 2013; Hanson and Atance, 2014), which limited their statistical power and the generalizability of their results. Furthermore, in most cases, narrative-based measures were used to assess MTT abilities in people with ASD. However, as previously discussed narrative difficulties are usually present in this clinical population, even if at different degrees. As such, the group differences in experimental task performance might merely reflect general difficulties in narrative rather than specific difficulties with EM and EFT (Gaesser et al., 2011; Race et al., 2011; Addis and Schacter, 2012). Several scholars (e.g., Lind and Williams, 2012; Terrett et al., 2013) have acknowledged such methodological bias. To the best of our knowledge, only one study assessing EM and EFT through linguistic tasks could claim that diminished experimental performance in remembering the past and imagining the future was not merely the result of reduced general narrative ability among participants with ASD (Lind et al., 2014). Compared to other studies, these findings along with the specific methodology used by the authors are of particular relevance as they allow to rule out that impairments in EM and EFT in individuals with ASD are related to linguistic deficits. More generally, these findings have implications for a theoretical issue relevant to the current investigation that concerns the relationship between time and narrative. In contrast to a well-known tradition according to which temporal projection is the product of narrative (e.g., Bruner, 1999, 1991; Dautenhahn, 2002), the study by Lind et al. (2014) suggests that the representation of time is independent from narrative abilities. These considerations are particularly relevant as they pave the way for the possibility that the ability to mentally travel in time might have effects on narrative processing.

### The Present Study

In light of these considerations, the present study aimed at investigating the relation between one component of MTT, namely EFT, and narrative production skills by analyzing storytelling abilities in a group of children with ASD. Specifically, we aimed to verify (1) whether impairments of EFT could be identified in a large cohort of children with high functioning ASD using a task with minimal narrative demands, such as the *Picture Book Trip* task adapted from Atance and Meltzoff (2005) and (2) whether impairments in EFT affect narrative generation skills in children with ASD. For the first point, based on the literature, we predicted that EFT would be impaired in only some children with ASD. Regarding the second point, based on our hypothesis about the relation between time and narrative, we expected that participants with ASD with impaired EFT would have lower skills of narrative discourse production than would those with normal EFT abilities.

## Materials and Methods

### Participants

One hundred and thirty-two Italian-speaking children aged between 6 and 11.11 years were included in this study. They formed two groups with comparable chronological age and IQ-Level (see **Table 1**). The first group was formed by 66 children with high functioning ASD and IQ level (as measured with the Raven’s Coloured Progressive Matrices; Raven, 1938) in the normal range recruited at the Bambino Gesù Children’s Hospital in Rome, Italy. The gravity of their symptomatology was assessed by administering the Autism Diagnostic Observation Schedule 2nd edition – ADOS-2 by Lord et al. (2013). Overall, the group of participants with ASD had a mean gravity score of 6.07 with a standard deviation of 1.66 ranging from 2 to 9.

**Table 1:** General data of the two groups of participants.

	ASD (*n* = 66)	TD (*n* = 66)
Age	8.14 (1.51) range: 6.00–11.07	8.23 (1.51) range: 6.00–11.11
Education	1^st^ – 5^th^ grade	1^st^ – 5^th^ grade
Gender distribution	Males = 59 (89.0%)	Males = 38 (58.0%)
IQ level	106.06 (14.34) range: 80–141	105.91 (10.95) range: 90–130
ADOS-2 gravity index	6.07 (1.66) range: 2–9	–

*Data are expressed as means, standard deviations, percentages, and ranges where appropriate. Legend: ASD, (children with) autism spectrum disorders; TD, (children with) typical development; ADOS-2, autism diagnostic observation schedule 2nd edition.*

The control group was formed by 66 children with typical development (TD). All of them performed within normal range on a series of tasks aimed at assessing their levels of non-verbal intelligence (Raven’s Progressive Matrices; Raven, 1938) and their verbal short-term and working memory (Non-Word Repetition subtest of the *Prove di Memoria e Apprendimento per l’Età Evolutiva* (PROMEA, Vicari, 2007); the forward and backward digit span’s subtests of the Wechsler Scales (Wechsler, 1993)]. Furthermore, they had average school performance. In a preliminary interview, their teachers confirmed that they had normal cognitive and learning development. According to school records and parents’ reports, none of them had a known history of psychiatric or neurological disorders, learning disabilities, hearing or visual loss.

This study was carried out in accordance with the recommendations of the Bambino Gesù Children’s Hospital committee. The protocol was approved by the Bambino Gesù Children’s Hospital committee. Parents released their written informed consent to the participants of their children to the study and to the treatment of the data in accordance with the Declaration of Helsinki.

### Methods

The participants with diagnosis of ASD were tested at the Bambino Gesù Children’s Hospital in Rome. The participants with TD were tested individually at school. The tasks included a cognitive assessment focusing on phonological short-term and working memory, EFT skills and narrative abilities.

#### Assessment of Phonological Short-Term and Working Memory

Several scholars (e.g., Suddendorf and Corballis, 2007, p. 307) have suggested that working memory abilities are involved in the generation of a future event. In fact, a study by Ferretti et al. (2018) on 135 children with typical development has shown that EFT highly correlates with measure of verbal working memory. For these reasons, all participants were administered tasks aimed at assessing their phonological short-term and working memory [Digit Span forward and backward subtests of the Wechsler (1993) Scales and the Non-Word Repetition Task of the PROMEA; Vicari, 2007].

In the Digit Span forward task, the child is asked to repeat in the correct order sequences of digits spoken by the examiner. The digits range from 1 to 9 and vary in length. The number of lists correctly repeated by the child represents the Digit Span forward score. In the Digit Span backward task, the child is asked to repeat each sequence in the reverse order. Finally, in the Non-Word Repetition Task of the PROMEA the child is required to repeat a list of 40 *non-words* that the examiner reads aloud hiding her/his labial movements. Each correct answer is assigned 1 point for a maximum of 40 correct repetitions.

#### Assessment of EFT: The Picture Book Trip Task

In the *Picture Book Trip* task adapted from Atance and Meltzoff (2005) each child was shown, one at a time, four colored pictures illustrating different destinations for a trip: a waterfall, a long road in a sandy desert, a mountain view, and a rocky stream. They were asked to describe each picture’s contents, and then were explicitly asked to imagine themselves in the scenarios at a future time point. For each of the four target pictures (e.g., *the long road*), the experimenter showed three different photographs, each representing a specific item that could be: (1) useful in the target scenario (i.e., a *bottle of water*); (2) completely useless in that scenario and not related to the scene (i.e., a *gift*), (3) semantically primed by the scenario (i.e., a *plant*). The *Picture Book Trip* task is a good measure of EFT as it is designed to specifically evoke thoughts about future states of the self. For example, if asked to imagine oneself walking down a long road in a sandy desert, people would likely anticipate the state of thirsty. Following the original study by Atance and Meltzoff (2005), we selected events that did not form part of children’s daily routine to minimize “script-based” responses, but which children are familiar with. This suggests that they were anticipating how the showed events could lead to the corresponding states. This claim is strengthened by the addition of a semantically primed item among the options that allows to exclude that the choice was an effect of an associative link to the presented scenario. For its robustness, the task from Atance and Meltzoff (2005) has been used both in children with typical development (e.g., Atance and Jackson, 2009; Ferretti et al., 2018) and in children with ASD (e.g., Hanson and Atance, 2014).

Children were asked which of these items they would need to bring with themselves (“Which of the objects portrayed in these pictures will you need to take with you in this trip?”). After choosing the selected item, they were invited to motivate their answers, explaining how the selected item would be useful in that scenario anticipating potential future needs (“Why will you need this in your trip?”). Children received 1 point for each item that had been correctly chosen (Identification Score, IS). One additional point was assigned whenever they could adequately motivate their choice showing that they had been able to project themselves to meet a potential future need (Motivation Score, MS). Noteworthy, in the original version of the task, the Motivation Score was derived from a linguistic analysis of the motivation produced by the child for his/her choice. Namely, the child received 1 point only if (s)he included in the motivation (1) a future term (e.g., going to, will, when) and (2) words that explicitly referred to internal feelings. However, in Italian future states can be expressed also with present tense (e.g., “Domani vado a casa” “^∗^Tomorrow, I go home”). For this reason, in our study, to avoid a potential linguistic bias, the motivation received 1 point if it correctly explained the choice regardless of the linguistic form used by the child. A recent study conducted by our group on 135 children with typical development has shown that the *Picture Book Trip* task still works as a measure of EFT also removing the criterion of future tense use as a marker of motivation score (Ferretti et al., 2018). An EFT Composite Score (EFT_CS) was derived by summing up these two scores (IS and MS; maximum 8).

#### Assessment of Narrative Skills

**FIGURE 1:**
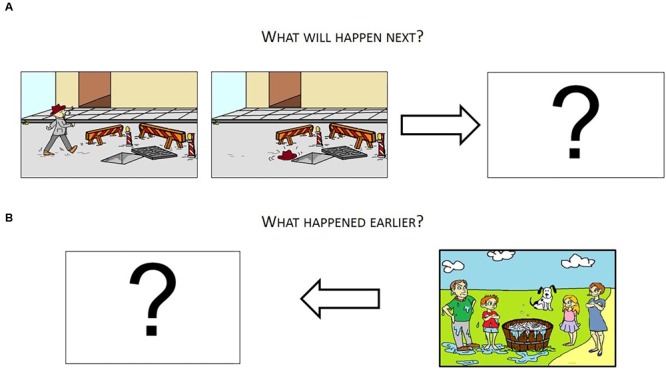
Cartoon stories designed to assess narrative production. **(A)** Shows future-generation condition (adapted from Pagni, 2011). **(B)** Shows past-generation condition (adapted from Ripamonti, 2013).

The ability to generate a coherent narrative discourse was assessed by administering two cartoon-story description conditions adapted from Pagni (2011) and Ripamonti (2013). Three cartoon-stories were designed to assess the children’s ability to generate a future episode in a narrative discourse (i.e., its conclusion) (see **Figure 1A**). The remaining three were used to assess their ability to generate a past episode (i.e., its antecedent) (see **Figure 1B**). For each condition, the first cartoon-story was used as a trial to allow children to get acquainted with the test.

In the future-generation condition, the experimenter arranged three sheets of paper on the table: the first two sheets contained colored drawings that portrayed the beginning of a story, while the third was blank. The experimenter began by describing the first picture (e.g., “Here is Mr. Mario. He is whistling as he walks down this road”; **Figure 1A**). She then asked the child to describe the second picture (e.g., “Mr. Mario fell down an open manhole”). While pointing at the blank sheet, the experimenter eventually asked the child to continue with the story by asking him or her, “What will happen next?” For each story, children could talk freely until they came to an end. If they did not finish the story, the examiners prompted them only once with a standardized question, “And then?”

In the past-generation task, the experimenter arranged two sheets on the desk. The first sheet was blank but the second contained a colored sketch. While pointing at it, the experimenter described the scene depicted in the second drawing (e.g., “There is a family in a garden with a dog wash tub and soapy water everywhere. Dad and son are wet and angry. Mom and daughter look at them amused. Their dog stares at the scene”; **Figure 1B**). While pointing at the blank sheet, the experimenter eventually asked the child to describe what could have likely happened earlier in this story by saying, “What happened earlier?” In this case, children were free to talk about the story until they felt they had provided the beginning of these stories. In case they did not finish the story, the examiners prompted them only once with a standardized question, “And then?”

To avoid poor performance due to short-term memory limitations, all pictures remained visible until participants had finished their descriptions. Each story was tape-recorded and subsequently transcribed verbatim; the transcriptions included phonological fillers, pauses, false starts, and extraneous utterances. These transcriptions were compared to obtain highly reliable texts for analysis. Discrepancies were discussed and resolved before the narratives were analyzed further. For all tasks, only the narrative samples produced by the child after the experimenter pointed at the blank sheets were included in the analyses. Each narrative was segmented into utterances, and the total number of utterances was assessed following criteria detailed in Marini et al. (2011). Accordingly, we adopted several criteria for utterance segmentation: acoustic, semantic, grammatical, and phonological ones. According to the acoustic criterion an utterance is an emission of phonemes delimited by pauses that can be easily identified. Let’s consider the following sequence: “this is a… (silent pause of 3 s) child.” In this case, since a clear pause can be perceived between the first chunk “this is a” and the second one “child,” the sequence can be segmented in two distinct utterances: /This is a… (5 s)/child/. According to the semantic criterion an utterance is a conceptually homogeneous piece of information—i.e., a proposition, defined as a semantic unit consisting of the main predicate with its arguments and all embedded predicates and argument(s) associated with it. Therefore, if there is not a sensible pause in the flow of speech, utterance boundaries can be identified whenever a proposition has been formulated and a new one is introduced. For example, the sequence “A man is walking on the road. A flower pot falls on his head” can be split in two distinct utterances: /A man is walking on the road/A flower pot falls on his head/ because the second utterance introduces a new proposition. Similar considerations can be applied to the following utterances: /A man is walking on/He is running on the sidewalk/ where the first proposition has not been completed and the second block provides a reformulation of the preceding one. According to the grammatical criterion for utterance segmentation, a block of words is an utterance when, in absence of clear pauses (acoustic criterion) and of propositional violations (semantic criterion), it forms a grammatically complete sentence (eventually also including subordinate clauses). In this case, even if long, the sequence can be considered a single utterance, as shown in the following example: /The man is walking on the sidewalk with a dog that looks very nice/. Nonetheless, if the speaker utters two coordinated sentences, these can be divided in two separate utterances: /The man is walking on the sidewalk/and a dog is following him/. This is because the utterance count is important for several derived measures capturing grammatical, cohesive and coherent aspects of linguistic processing. According to the phonological criterion if a word is interrupted (i.e., there is a false start), then the utterance is considered abruptly interrupted as well. For example, in the following sequence: /and she is ca-/stroking his d-/his d-/the dog of the man/ four distinct utterances can be identified and the first three end with a false start (ca-, d-, d-, respectively). The segmentation procedure was performed independently by three trained raters on 40 narratives selected randomly. The segmentation procedures resulted in substantial agreement, as the raters reached an inter-coder reliability level of *k* > 0.91.

To evaluate the narratives’ global coherence, each utterance was analyzed in five main aspects: elements that were included in the pictures’ stimuli, new elements not originally included in the pictures’ stimuli, direct causal links, indirect causal links, and new elements without causal links. An utterance received 1 point if it contained elements (e.g., characters, objects, and actions) already included in the pictures’ stimuli. For example, the following utterance /*il cappello rimane per terra*/ (“the hat is on the ground”) was evaluated as containing an element already present in the stimuli (see **Figure 1A**). The percentage of elements included in the pictures’ stimuli was calculated by dividing the total number of such elements by the number of utterances and then multiplying by 100. Higher values indicate the child’s inability to project in time: the more the child produced utterances describing elements contained in the picture, the more he or she demonstrated being stuck on the image stimulus. An utterance received 1 point if it contained new elements that were not included in the starting pictures. As for the following example, */va nelle fogne/* (/“he goes in the sewers”/), the utterance received 1 point as it contained elements that were not present in the image stimulus (see the **Figure 1A**). The percentage of new elements was measured by dividing the total number of new elements by the number of utterances and then multiplying by 100. Higher values represent the child’s ability to project in time. In this case, the more the child produced utterances introducing new elements not contained in the picture, the more (s)he demonstrated the ability to detach from the image stimulus.

Each utterance was also analyzed by considering the causal connections it had with other utterances of the story. Two causal connections were evaluated: direct causal links and indirect causal links. Direct causal links were assessed as follows: an utterance received 1 point when it had a direct causal connection with the events illustrated in the stimuli. A causal connection is established between a pair of events when the criterion of necessity is satisfied (Trabasso and Sperry, 1985; Diehl et al., 2006; Sah and Torng, 2015). Necessity was tested by using the counterfactual argument of the form: if not A, then not B. That is, if Event A had not happened in the story, then Event B would not have happened. Accordingly, Event A is a cause of or a condition for Event B, and the two events are considered causally connected. For indirect causal links, an utterance received 1 point if it was not directly connected to the events in the images stimuli but was connected to other sentences that the child had previously stated. In the following sample, */va nelle fogne/non riesce a risalire perché è pericoloso/ e continua/ e trova una scala per l’uscita/* (“*/*he goes in the sewers/he cannot get out because it is dangerous/and he goes on/and he finds a stair to go out/”) the first utterance was evaluated as containing a direct causal link (as well as a new element) whereas the second, the third and the fourth were considered as containing indirect causal links (as well as new elements) connected to the information provided in the previous sentence (see **Figure 1A**). The sum of direct and indirect causal links was used to calculate a causal link score measured by dividing that sum by the number of utterances and then multiplying by 100. Higher values indicate the child’s ability to connect episodes of the story. Our hypothesis was that temporal projections and the ability to connect the events of a story causally were both necessary for the generation of coherent narratives. For this, we considered high values in new elements and causal links as indicative of global coherence of the stories. Overall, these two measures contributed to characterize in positive terms the narrative global coherence.

We also looked at the errors children were producing when telling the stories in order to have a measure of the degree of incoherence of the narratives. Specifically, we evaluated errors of global coherence by considering tangential and conceptually incongruent utterances, defined in terms of utterances with new elements without causal links. An utterance was considered tangential when it contained a derailment in the flow of discourse with respect to the information already provided in a preceding utterance. Finally, an utterance was considered conceptually incongruent when it included concepts not directly addressed by the stimulus. For example, in the sequence “/*aveva fatto cadere il cappello via verso la casa/aveva costruito un garage/e quindi ci aveva costruito qualcosa*/” (“/He dropped the hat toward the house/he built a garage/then, he built something/”) (see **Figure 1A**) the second and the third utterances were evaluated as conceptually incongruent and defined as utterances with new elements without causal links. A percentage of global coherence errors was calculated by dividing the number of global coherence errors by the number of utterances and multiplying this value by 100.

Coding was carried out independently by three trained coders. Kappa coefficients assessing inter-coder reliability exceeded 0.95 for all five measures (elements included in the pictures’ stimuli, new elements, direct causal links, indirect causal links and new elements without causal links) on 40 narratives randomly selected.

## Results

### Analysis of Phonological Short-Term and Working Memory Skills of the Two Groups

The group-related differences on the assessment of the children’s cognitive skills were analyzed with *t*-tests with group (i.e., ASD vs. TD) as fixed factor and the two cognitive measures (i.e., scores at the digit span forward and backward subtests of the WISC) as dependent variables. The level of statistical significance was set at *p* < 0.025 (0.05/2 dependent variables) after Bonferroni correction for multiple comparisons. As shown in **Table 2**, the two groups differed on their performance at the Forward Digit Span subtest of the WISC [*t*_(129)_ = -4.421; *p* < 0.001; Cohen’s *d* = 0.78] but performed similarly at the Backward Digit Span subtest of the WISC [*t*_(129)_ = -1.135; *p* = 0.258; Cohen’s *d* = 0.20].

**Table 2:** Phonological short-term and working memory in the two groups of participants.

	ASD	TD
	*M* (*SD*) [min-max]	*M* (*SD*) [min-max]
Digit forward^∗^	5.80 (1.43) [3–10]	6.99 (1.63) [3–11]
Digit backward	3.49 (1.61) [0–8]	3.77 (1.19) [2–6]

*Data are expressed as means, standard deviations, and ranges. ASD, (children with) autism spectrum disorder; TD, (children with) typical development.*

*Asterisks (^∗^) show when group-related differences were significant after Bonferroni correction for multiple comparisons (p < 0.025).*

### Analysis of Episodic Thinking and Narrative Skills in Children With ASD and Children With TD

As the two groups differed on their performance at the Forward Digit Span subtest of the WISC, we aimed to assess the presence of group-related differences on EFT considering the potentially confounding role of phonological short-term memory. The relationship between performance at the Forward Digit Span task and the measures of EFT and narrative generation was investigated by using Pearson product-moment correlation coefficient on the whole sample of participants. Significant positive correlations were found between the Forward Digit Span scores, the EFT Score (*r* = 0.375; *p* < 0.001), the elements included in the pictures’ stimuli (*r* = -0.204; *p* < 0.020), the new elements (*r* = 0.380; *p* < 0.001), the causal links score (*r* = 0.407; *p* < 0.001) and the percentage of global coherence errors (*r* = -0.221; *p* < 0.012). For this reason, the group-related differences on the assessment of the participants’ EFT skills and the four narrative scores were analyzed by performing a series of Univariate ANCOVAs with group (i.e., ASD vs. TD) as fixed factor, the EFT Score, the elements included in the pictures’ stimuli, the new elements, the causal links scores and the percentage of global coherence errors as dependent variables, and the participants’ performance at the Forward Digit Span subtest of the WISC as covariate. The level of statistical significance was set at *p* < 0.001 (0.05/5 dependent variables) after Bonferroni correction for multiple comparisons (see **Table 3**). The analysis revealed that the participants with ASD scored significantly lower than children with TD on measures assessing EFT [*F*(1,127) = 29.552, *p* < 0.001, $\eta_{p}^{2}$ = 0.187], the presence of new elements [*F*(1,127) = 29.417, *p* < 0.001, $\eta_{p}^{2}$ = 0.188], and the causal link score [*F*(1,127) = 39.332, *p* < 0.001, $\eta_{p}^{2}$ = 0.236]. However, the two groups did not differ on the referral to elements portrayed in the pictures (elements included in the pictures’ stimuli: [*F*(1,127) = 0.143, *p* = 0.706, $\eta_{p}^{2}$ = 0.001] and in the percentage of errors of global coherence [*F*(1,127) = 8.119, *p* = 0.005, **$\eta_{p}^{2}$** = 0.060].

**Table 3:** Performance of the two groups on tasks assessing Episodic Future Thinking skills and narrative scores (i.e., elements included in the pictures’ stimuli, new elements causal links, and global coherence errors).

	ASD	TD
	*M* (*SD*) [min-max]	*M* (*SD*) [min-max]
EFT^∗^	5.14 (2.01) [1–8]	7.09 (1.21) [5–8]
Elements included in the pictures’ stimuli	16.24 (11.65) [0–50]	15.43 (6.51) [2–30]
New elements^∗^	42.77 (14.49) [15–71]	58.89 (12.77) [24–87]
Causal links^∗^	31.34 (14.36) [5–64]	47.39 (8.85) [22–65]
Global coherence errors	4.76 (8.14) [0–43.84]	0.80 (3.52) [0–25.00]

*Data are expressed as means, standard deviations, and ranges. ASD, (children with) autism spectrum disorder; TD, (children with) typical development; EFT, episodic future thinking score.*

*Asterisks (^∗^) show when group-related differences were significant after Bonferroni correction for multiple comparisons. Elements included in the pictures’ stimuli: higher values indicate the child’s inability to project in time. New elements: higher values represent the child’s ability to project in time. Causal links: higher values indicate the child’s ability to connect each other the episodes of the story. % Global Coherence Errors: higher values indicate the child’s inability to establish coherent links among the utterances that form each storytelling*.

Potential group-related differences on the two narrative conditions (i.e., past- or future-generation condition) were explored by performing additional Univariate ANCOVAs with group (i.e., ASD vs. TD) as fixed factor, the elements included in the pictures’ stimuli, the new elements, the causal links scores and the percentage of global coherence errors as dependent variables, and the participants’ performance at the Forward Digit Span subtest of the WISC as covariate separately for the two narrative conditions. For each condition, the level of statistical significance was set at *p* < 0.013 (0.05/4 dependent variables) after Bonferroni correction for multiple comparisons. In the future-generation condition, the participants with ASD produced fewer new elements [*F*(1,127) = 17.074, *p* < 0.001, $\eta_{p}^{2}$ = 0.118] and causal links [*F*(1,127) = 29.079, *p* < 0.001, $\eta_{p}^{2}$ = 0.185] than controls. However, the two groups did not differ on the referral to elements portrayed in the pictures [*F*(1,127) = 0.143, *p* = 0.706, $\eta_{p}^{2}$ = 0.001] and in the percentage of errors of global coherence [*F*(1,127) = 5.040, *p* = 0.026, $\eta_{p}^{2}$ = 0.038].

In the past-generation condition, the participants with ASD produced fewer new elements [*F*(1,127) = 16.589, *p* < 0.001, $\eta_{p}^{2}$ = 0.116], causal links [*F*(1,127) = 21.699, *p* < 0.001, $\eta_{p}^{2}$ = 0.146] and more errors of global coherence [*F*(1,127) = 6.903, *p* < 0.010, $\eta_{p}^{2}$ = 0.052]. However, the two groups did not differ on the referral to elements portrayed in the pictures [*F*(1,127) = 0.016, *p* = 0.898, $\eta_{p}^{2}$ = 0.001].

In order to control for the potential difficulty of patients with ASD more on one narrative condition (past- or future-generation condition) than the other, a series of paired sample *t*-tests were conducted for the two groups separately on the narrative production variables (i.e., the elements included in the pictures’ stimuli, the new elements, the causal links scores and the percentage of global coherence errors). Also in this case, the level of statistical significance was set at *p* < 0.013 (0.05/4 dependent variables) after Bonferroni correction for multiple comparisons. These analyses showed the absence of any condition related difference within this group.

### Further Analysis of Narrative Skills in ASD

In order to further explore the possibility that a subgroup of children with ASD might experience significantly impaired future thinking skills, the group of participants with ASD was split in two subgroups according to their performance on the task of EFT. As normative data for this task are currently not available, we considered a score of 5–8 at the *Picture Book Trip task* as normal as this was the range observed in the control group. This allowed us to identify a subgroup of participants with ASD who had low and one with high performances on this task. This analysis revealed that 25 participants with ASD had significant difficulties in EFT, whereas 41 of them did not experience such difficulties. The two subgroups of individuals with ASD and the group of typically developing children had comparable chronological age [*F*(2,131) = 0.176, *p* = 0.839] and IQ level [*F*(2,131) = 1.470, *p* = 0.234]. Importantly, the two subgroups of participants with ASD did not differ in the ADOS-2 Gravity Index either [*t*_(63)_ = 0.913; *p* = 0.365] (see **Table 4**). Finally, the three groups differed on the Digit Span Forward ([*F*(2,130) = 12.044, *p* < 0.001]) with the two subgroups of participants with ASD not differring from each other on this measure (*p* = 0.112) but participants with lower EFT skills and children with higher EFT skills both performing worse than children with TD (*p* < 0.001, and *p* < 0.012, respectively).

**Table 4:** Cognitive profile of the two groups with ASD.

	ASD LowEFT (*n* = 25)	ASD HighEFT (*n* = 41)
	*M* (*SD*) [min–max]	*M* (*SD*) [min–max]
Age	8 (1.42) [6–10]	8.22 (1.57) [6–11]
IQ level	103.34 (14.19) [85–130]	108.72 (14.21) [80–141]
Ados 2 gravity index	6.30 (1.46) [3–8]	5.92 (1.78) [2–9]
Digit forward^∗^	5.34 (1.23) [3–8]	6.10 (1.48) [3–10]
EFT^∗^	3.07 (1.09) [1–4]	6.55 (1.10) [5–8]

*Data are expressed as means, standard deviations, and ranges. ASD LowEFT, children with ASD who did not perform well on the EFT task; ASD HighEFT, children with ASD who performed well on the EFT task; EFT, episodic future thinking score; ADOS-2, autism diagnostic observation schedule, 2nd edition. Please, note that means, standard deviations and ranges of the group of children with typical development are available in **Table 3***.

*Asteriks (^∗^) show when group-related differences were significant after Bonferroni correction for multiple comparison.*

Potential group-related differences between the three groups of participants (ASD children who had a good performance on EFT task, those who had difficulties on this task and the group of children with typical development) were assessed with a series of Univariate ANCOVAs with group as fixed factor, the four narrative scores as dependent variables and performance on the Digit Span Forward task as a covariate. The level of statistical significance was set at *p* < 0.013 (0.05/4 dependent variables) after Bonferroni correction for multiple comparisons (see **Table 5**). Group-comparisons were performed by applying the Least Significant Difference (LSD) t test with Bonferroni adjustment. Significant group-related differences were found for all of these measures: elements included in the pictures’ stimuli [*F*(2,130) = 9.867, *p* < 0.001; $\eta_{p}^{2}$ = 0.135], new elements [*F*(2,130) = 22.566, *p* < 0.001; $\eta_{p}^{2}$ = 0.246], the causal link score [*F*(2,130) = 30.509, *p* < 0.001; *$\eta_{p}^{2}$* = 0.326], and the percentage of errors of global coherence [*F*(2,130) = 6.229, *p* < 0.003; $\eta_{p}^{2}$ = 0.090]. The group comparisons showed that those ASD participants who performed lower on the *Picture Book Trip* task produced more elements included in the pictures’ stimuli than participants with ASD who performed normal on the same task (*p* < 0.001) who, in turn, produced the same number of elements included in the pictures’ stimuli as control participants (*p* = 0.082). Furthermore, those ASD participants who performed lower on the *Picture Book Trip* task produced fewer new elements (*p* < 0.007) and causal links (*p* < 0.001) than participants with ASD who performed normal on the *Picture Book Trip* task who scored lower than controls (all *p*s < 0.001). Finally, those ASD participants who performed lower on the *Picture Book Trip* task produced the same number of global coherence errors as participants with ASD who performed normal on the EFT task (*p* = 0.132) but only those who performed lower on the *Picture Book Trip* task produced a significantly higher number of such violations with respect to controls (*p* < 0.002).

**Table 5:** Narrative scores in the three groups of participants.

	ASD LowEFT	ASD HighEFT	TD
	*M* (*SD*) [min–max]	*M* (*SD*) [min–max]	*M* (*SD*) [min–max]
Elements included in the pictures’ stimuli^∗^	22.76 (14.46) [2–50]	12.17 (7.06) [0–33]	15.43 (6.51) [2–30]
New elements^∗^	35.54 (14.96) [15–71]	47.28 (12.35) [24–70]	58.89 (12.77) [24–87]
Global coherence errors^∗^	6.95 (9.04) [0–43.84]	3.39 (7.32) [0–42.50]	0.80 (3.52) [0–25.00]
Causal links^∗^	23.46 (13.50) [5–52]	36.26 (12.71) [5–64]	47.39 (8.85) [22–65]

*ASD LowEFT, children with ASD who did not perform well on the EFT task; ASD HighEFT, children with ASD who performed well on the EFT task; TD, children with typical development*.

*Asterisks (^∗^) show when group-related differences were significant after Bonferroni correction for multiple comparison.*

In order to control for the potential difficulty of the two subgroups of patients with ASD more on one narrative condition than the other (i.e., past- or future-generation condition), a series of paired *t*-tests were conducted for the two groups separately on the narrative production variables (i.e., the elements included in the pictures’ stimuli, the new elements, the causal links scores and the percentage of global coherence errors). Also in this case, the level of statistical significance was set at *p* < 0.013 (0.05/4 dependent variables) after Bonferroni correction for multiple comparisons. These analyses showed the absence of any condition related difference for the group of participants who performed well on the EFT task. However, those who scored lower on that task produced significantly more new elements (*p* < 0.001) and causal links (*p* < 0.009) on the future-generation condition.

### An Inspection of the Relationship Between Measures of EFT and Narrative Generation

The relationship between the measures of EFT and narrative generation was investigated by using Pearson product-moment correlation coefficient on the two groups of participants (children with ASD and children with typical development).

In children with typical development, no significant correlation was found between the measure of EFT and the four narrative variables: elements portrayed in the pictures (*r* = -0.076; *p* = 0.546); new elements (*r* = 0.040; *p* = 0.754); causal links (*r* = 0.133; *p* = 0.291); global coherence errors (*r* = -0.051; *p* = 0.685). On the contrary, in children with ASD significant correlations were found for the EFT Score and the number of elements included in the pictures’ stimuli (*r* = -0.421; *p* < 0.001), the new elements (*r* = 0.379; *p* < 0.002), the causal links’ score (*r* = 0.425; *p* < 0.001), and the percentage of errors of global coherence (*r* = -0.247; *p* < 0.045).

A further inspection of such correlations in the two subgroups of participants with ASD (with low or high EFT skills), showed a different picture. Indeed, no significant correlation was found in any of the two groups. In children with ASD and low EFT skills the EFT Score did not correlate with the number of elements included in the pictures’ stimuli (*r* = 0.001; *p* = 0.997), the new elements (*r* = -0.020; *p* = 0.924), the causal links’ score (*r* = -0.004; *p* = 0.984) or the production of errors of global coherence (*r* = -0.090; *p* = 0.667). Similarly, in children with ASD and high EFT skills no significant correlations were detected between the EFT Score and the number of elements included in the pictures’ stimuli (*r* = -0.197; *p* = 0.216), the new elements (*r* = 0.180; *p* = 0.259), the causal links score (*r* = 0.200; *p* = 0.211), and the percentage of global coherence errors (*r* = -0.140; *p* = 0.381).

## Discussion

The current study analyzed the relation between a specific component of MTT, namely EFT, and narrative generation skills in a group of school-aged children with high-functioning ASD. To avoid a potential severity bias, children with different severity levels of autistic symptomatology were included in the experiment. The performance of the group of children with a diagnosis of ASD was compared to that of a group of children with typical development matched on age, level of formal education, and IQ. EFT was assessed by administering a task with minimal narrative demands, whereas storytelling skills were assessed with two tasks requiring children to generate future or past episodes in a narrative discourse. Since our hypothesis was that the ability to mentally project in time and the ability to causally connect the events of a story are both involved in the construction of global coherence, the analyses of the narratives focused on the count of elements included in the pictures’ stimuli, new elements introduced, causal links (both direct and indirect), and errors of global coherence (i.e., new elements that were tangential or incongruent with the storyline).

Regarding the first prediction of our study, the results showed – consistent with the expectations – that EFT skills were impaired in a subgroup of children with ASD. On the contrary, the second prediction was only partially confirmed by our findings. On the one hand, in children with ASD significant correlations were found between EFT and the four narrative measures (i.e., elements included in the pictures’ stimuli, new elements, causal links, and errors of global coherence). On the other hand, we did not find a correlation between EFT score and the narrative measures when the group of ASD was split into two subgroups (namely, the children with low EFT skills and those with high EFT skills). Interestingly, however, looking at the groups’ differences, the subgroup of children with ASD who obtained lower scores on the EFT task generated stories containing a higher percentage of elements included in the pictures stimuli and fewer new elements and causal links with respect to both ASD participants with higher EFT skills and controls. Furthermore, the subgroup of participants with ASD who performed lower on the EFT task produced more errors of global coherence than controls.

When the two narrative conditions (future-generation vs. past-generation) were considered separately, interesting group-related differences emerged. Namely, in the past-generation condition, the group of participants with ASD produced fewer new elements and causal links than the group of participants with typical development, but more errors of global coherence. In the future-generation condition children with ASD produced fewer new elements and causal links than controls but the two groups did not differ both in the number of elements included in the picture stimuli and in the percentage of errors of global coherence. No differences were found between the two narrative conditions in the ASD group: participants with ASD performed equally both in past- and future-generation condition. However, an unexpected finding is that the subgroup of ASD with lower EFT skills performed better in the future-generation condition. In the future-narrative task, indeed, that subgroup produced more new elements and causal links compared to the subgroup of ASD with higher EFT skills. Overall, these results partially support the hypothesis of a relation between temporal navigation skills and the ability to generate coherent narratives. These findings have important practical and theoretical implications.

Our results support the possibility of an impairment in episodic foresight in children with ASD. This is in line with the few studies assessing EFT in children with ASD (Jackson and Atance, 2008; Terrett et al., 2013; Hanson and Atance, 2014; Marini et al., 2016). However, differing from previous investigations because of the large cohort of children included in the present study, our results show that not all individuals with ASD might manifest difficulties in EFT. The possibility that EFT is not homogeneously impaired in individuals with ASD might explain the apparently incongruent results by Crane et al. (2013) and, at least partly, by Hanson and Atance (2014). As mentioned in Section “Episodic Memory and Episodic Future Thinking in Autism,” Crane et al. (2013) did not find any group-related difference between adults with ASD and neurotypical participants on a sentence completion task employed to generate past and future event descriptions. Hanson and Atance (2014) assessed episodic foresight in children with ASD using a battery of five EFT tasks. In that study, a group of children with ASD showed difficulties only on some of them, namely the sequencing task (judging the temporal distance of short- and long-term future life events; Busby Grant and Suddendorf, 2009), the Grocery/Beach task (reporting verbal plans for going to the grocery store and the beach; Hudson et al., 1995), and the Picture Book task (Atance and Meltzoff, 2005), which is similar to that used in the present study. Hanson and Atance (2014) did not find any group-related differences between children with ASD and children with typical development on the Tomorrow task (explaining what they will and will not be doing tomorrow; Busby and Suddendorf, 2005) or the Zoo task (placing a toy camera in a zoo setup, making inferences using temporal order information; McColgan and McCormack, 2008). The absence of differences in some tasks might depend on a methodological bias but also on the inclusion of a heterogeneous group of ASD participants containing both impaired and normal EFT skills.

The second relevant finding of our study was that the whole group of children with ASD produced less coherent narratives than the group of children with typical development. This is consistent with other investigations reporting impaired narrative skills in individuals with ASD, mainly regarding the qualitative aspects of a story. Indeed, from a quantitative point of view (e.g., narrative length, structure, and complexity), the narratives of individuals with ASD showed few differences compared to the stories produced by individuals in control groups (e.g., Loveland et al., 1990; Norbury and Bishop, 2003; Diehl et al., 2006). On the contrary, from a qualitative point of view (e.g., representation of the gist of the story and organization of coherent chains of events), differences were more noticeable (e.g., Baron-Cohen et al., 1986; Loveland et al., 1990; Losh and Capps, 2003; Barnes and Baron-Cohen, 2012). For example, Sah and Torng (2015) found that stories of children with ASD did not differ from those of a control group on basic narrative measures, such as narrative length and variety of words. However, when stories were analyzed from a qualitative point of view, the narratives of children with ASD were less causally connected and less coherent. Consistently, our results show that when narratives are assessed from a qualitative point of view (e.g., focusing on the ability to relate a set of events sequentially organized in time), individuals with ASD tend to produce less coherent narratives than the control group. A similar result was found also when the two narrative conditions (i.e., past- and future-generation) were analyzed separately. The group of participants with ASD produced fewer new elements and causal links both in the past-condition and future-condition: their ability to construct coherently organized narratives was similarly impaired both when they were asked to generate a past-oriented and a future-oriented story. Moreover, the analyses showed the absence of any condition related difference within the group of ASD. A result of this kind suggests that children with ASD may show a core problem in the generation of narrative independently from the specific temporal orientation (i.e., past or future). This observation could be consistent with those hypotheses that link the ability to remember/foresee past/future events to a similar construction process (Hassabis and Maguire, 2007, 2009). However, further findings of the current study suggest that past and future constructions might not completely overlap. In fact, in the past-condition the group of ASD generated more errors of global coherence than in the future-condition. With regard to the specific task employed in the present study, producing past-oriented stories appeared to be more difficult for this clinical population. A similar difference in the two narrative conditions was observed also when the two subgroups of children with ASD were analyzed separately. The participants with ASD with lower EFT skills produced more new elements and causal links in the future-condition compared to the past-condition. This represents an unexpected finding. In fact, several studies suggested that in neurotypical population imagining future events is more cognitively effortful than remembering past events (see Perrin and Michaelian, 2017, p. 229). For example, Okuda et al. (2003) reported greater activity in left parahippocampal areas during tasks assessing EFT than in tasks assessing EM and related such result to the fact that thinking about future events requires a significant reactivation of episodic experiences that need to be recombined in order to construct a meaningful future episode. Similarly, when it comes to individuals with ASD Lind and Bowler (2010) found that adults with such disorder performed better in recalling past events as opposed to simulating future ones. For this, the unexpected result we found is worthy being investigated in future research.

Interestingly, the narrative measures in the whole group of children with ASD correlated with the scores they obtained on the *Picture Book Trip* task. This finding is particularly relevant as allows to maintain that the ability to project in time plays a role in the narrative skills of this group. Whilst this result supports our general hypothesis of a relation between time and narrative, the specific prediction that the performance on the narrative task of participants with ASD with impaired EFT would have been correlated with the score on the *Picture Book Trip* task was not confirmed. When we considered the two subgroups of children with ASD separately, indeed, we did not find a correlation between the narrative variables and the EFT score. It is worthy highlighting, however, that we noticed an interesting trend suggesting that a relation between temporal representation and narrative is in place: the subgroup of ASD who obtained lower scores on the EFT task performed significantly worse on the narratives measures than the group of participants with ASD with higher EFT skills or the group of participants with typical development. More specifically, the subgroup of children with ASD with impaired EFT skills produced narratives with fewer new elements and causal links. Therefore, this might indicate that the group of participants with ASD showing low EFT skills was less efficient in detaching from the images while constructing new detailed and connected episodes of the stories.

Our findings suggest that the idea that the cognitive systems involved in temporal projection might play a role in the processing of narrative coherence is still plausible. To this regard, the results of our behavioral outcomes are consistent with those of a neuropsychological study conducted by Race et al. (2015) examining the discourse of amnesic patients with medial temporal lobe damage. In that study, the authors showed that the hippocampus, one of the brain structure crucially involved in temporal projection (see Addis and Schacter, 2012; Corballis, 2015), supports the integration of narrative elements into coherent discourse when constructing complex verbal accounts. In this perspective, the time factor might represent one of the elements characterizing the processing of global coherence. In fact, it should be noted that the processing of narrative is a complex and multifaceted ability that rests on several intertwined cognitive components. Among others, a leading role is played by the ability to think imaginatively (Oatley, 2011). As reduced imagination has been reported in individuals with ASD (e.g., Craig and Baron-Cohen, 1999; Crespi et al., 2016), it is likely that some of their impairments in the narrative domain as well as in temporal projection can be affected by such a difficulty in imaging novel events. However, the specific narrative task used in the current study required not only to imagine novel events but also to process sequences of events by creating causal and temporal connections between them. This represents a crucial point. While imagination *per se* does not have an inherent temporal character, our task specifically elicited a temporal form of imagination.

The specific constraints of the current narrative task, namely the requirement to create novel events, lead to a further issue. Many investigations aimed at analyzing the narrative abilities in individuals with ASD (e.g., Tager-Flusberg, 1995; Tager-Flusberg and Sullivan, 1995; Capps et al., 2000; Losh and Capps, 2003; Norbury and Bishop, 2003; Sah and Torng, 2015) used picture sequences such as the wordless picture story books, e.g., *Frog, where are you* (Mayer, 1969) and *Frog on his own* (Mayer, 1973). In these investigations, participants were shown a sequence of pictures and were asked to tell a story based on this. However, several studies have shown that having the aid of looking at the sequence of pictures reduces the cognitive demands of storytelling for the participants, and therefore increases the length and complexity of narratives (e.g., Crais and Lorch, 1994; Gummersall and Strong, 1999). Furthermore, in generating a narrative based on a sequence of pictures, the narrator is required to understand the images and recognize the need to provide a sequenced interpretation of them (Stirling et al., 2014). For this reason, the wordless picture story book can be mainly considered as a comprehension task, with the narrative generation being a byproduct of the understanding of the pictures. Instead, in the narrative task used in the current study the participant is required to construct a novel creative story, with two additional constraints, i.e., generating past and future oriented stories. For these reasons, the current tasks require additional demands than the wordless picture story books.

Overall, these considerations have implications for a more general theoretical issue. The idea underlying the present work is that the time factor (i.e., the human ability to mentally project backward and forward in time) represents one of the abilities involved in the processing of global coherence—the property responsible for the construction of the plotline of a story. The relation between time and narrative has been widely investigated in Bruner’s cultural psychology (Bruner, 1999, 1991). From Bruner’s perspective, the main idea is that temporal projections are the product (rather than the condition) of narrative. Following Bruner, Dautenhahn (2002, p. 107) maintained that narrative allows humans to extend their temporal horizon, allowing them to travel forward and back in time. In a perspective of this kind, narrative and language are strictly tied to each other. Several scholars who adhere to Bruner’s psychology also adhere to the *language-first hypothesis* (Scalise-Sugiyama, 2005; Hutto, 2007; Gallagher and Hutto, 2008), the idea that language represents the constitutive element (and the evolutionary precondition) of narrative. From this perspective, temporal projection relies on narrative, that is, the ability to mentally travel in time is considered the product of language, the result of its grammatical complexity. This cultural psychology tradition has also influenced research on narrative in individuals with ASD. Quoting Bruner (1991), Losh and Capps (2003; Capps et al., 2000) suggested that narrative impairments of children with ASD were due to their poor grammatical skills, specifically with their difficulty to manage the complexity of syntax. According to the authors, this difficulty had effects on the narrative level, given that “the use of complex syntax is an important linguistic tool that enables narrators to mark temporal and causal distinctions between story events” (Capps et al., 2000, p. 201).

The idea that global coherence could be explained in reference to grammatical elements—that coherence can be reduced to cohesion—has been proposed by many scholars (Halliday and Hasan, 1976; Reinhart, 1980; see for a criticism Giora, 1985; Cosentino et al., 2013). In opposition to this idea, our view is that global coherence is primarily a cognitive phenomenon rather than a linguistic one. Indeed, as Givon (1995) suggested, global coherence is a mental phenomenon strongly tied to narrative production and comprehension processes. In the light of these considerations, we maintain that if the time factor represents one of the elements characterizing the processing of global coherence, then the processing of narrative has to be connected to the human ability to project backward and forward in time. The results of the present study suggest that such an ability is involved in the construction of a story’s plotline. Therefore, contrary to the Bruner tradition (Bruner, 1991, 1999; Dautenhahn, 2002), such results lead us to maintain that narrative is the product, rather than the condition, of the ability to mentally travel in time.

## Conclusion

The results of this study lead us to believe that the difficulties in storytelling shown by children with ASD are partly tied to their inability to mentally project in time. In light of these findings, it is possible to propose an integration of the fractionable model advanced to account for the narrative deficits of individuals with ASD. In addition to the impairments of theory of mind, central coherence and executive functions, the narrative patterns of individuals with ASD can be interpreted also with regard to an impairment in the ability to mentally travel in time. However, it is worth noting that other abilities might be involved in the processing of narrative, including more general skills as imagination. The specific contribution of such skills in narrative construction deserves to be investigated in future studies. The general conclusion drawn from these data is that the ability to tell stories relies on cognition more than cognition relies on language. In this sense, it is certainly true that, through stories, humans can extend themselves in the time and space dimensions, but it is primarily true that without the time navigation capabilities, humans could never have had the ability to tell stories.

## Author Contributions

FF planned the study, adapted the original tests, supervised the recruitment of the participants, and wrote Sections “Introduction,” “Narrative Global Coherence as the Construction of Causal Chains,” “Acknowledgment of the Time Factor in Narrative Processing,” “Discussion,” “Conclusion.” IA contributed to the adaptation of the original tests, recruited the participants, administered the tasks, and wrote Sections “Episodic Memory, Episodic Future Thinking and Their Relationships,” “Episodic Memory and Episodic Future Thinking in Autism,” “The Present Study,” “Participants,” “Methods,” “Discussion.” AC contributed to the adaptation of the original tests, recruited the participants, administered the tasks, and wrote Sections “Methods” and “Discussion.” SN contributed to the adaptation of the original tests, recruited the participants, and administered the tasks. GV and SV supervised the recruitment of the participants and the administration of the tasks and contributed to the interpretation of the data. RM recruited the participants, administered the tasks, and contributed to the interpretation of the data. AM supervised the recruitment of the participants and the administration of the tasks, ran the statistics, wrote the Section “Methods” and “Results” and contributed to the “Discussion”.

## Conflict of Interest Statement

The authors declare that the research was conducted in the absence of any commercial or financial relationships that could be construed as a potential conflict of interest.
